# Development of a COVID-19 Vaccination Anxiety Scale to measure COVID-19 vaccine anxiety in Japanese adults

**DOI:** 10.1371/journal.pone.0330146

**Published:** 2026-07-07

**Authors:** Time Fukuda, Ranno Haruyama, Yuto Tanaka, Shuma Natori, Kohei Koiwa, Koubun Wakashima

**Affiliations:** 1 Graduate School of Education, Tohoku University, Sendai, Japan; 2 Graduate School of Human Sciences, Osaka University, Osaka, Japan; 3 Graduate School of Education, Hokkaido University of Education, Hokkaido, Japan; Yeditepe University, TÜRKIYE

## Abstract

Although COVID-19 vaccines are widely available and effective, vaccination anxiety remains a significant public health challenge that is distinct from behavioral vaccine hesitancy. While various scales measure hesitancy, few specifically capture the multifaceted psychological anxiety regarding COVID-19 vaccines, and none have been validated for the Japanese population. To directly quantify this affective component, this study aimed to develop and validate the COVID-19 Vaccination Anxiety Scale for Japanese adults. A two-wave web-based survey was conducted with 500 Japanese adults. Participants completed the newly culturally adapted 25-item scale alongside validation measures assessing fear of COVID-19, vaccination readiness, conspiracist beliefs, and perceived vulnerability to disease. Test–retest reliability was evaluated after a four-week interval. Factor analyses revealed a three-factor structure: Anxiety Related to Vaccine Confidence, Emotional Symptoms of Anxiety, and Anxiety about Infection after Vaccination. The scale showed good internal consistency and high test–retest reliability, and multigroup analyses suggested approximate comparability of the three-factor structure across gender. Construct validity was supported through significant, theoretically aligned correlations with the external criteria. Specifically, vaccination anxiety was strongly associated with lower vaccination readiness, higher fear of infection, and greater conspiratorial beliefs. These findings highlight that vaccination anxiety is a complex emotional response that requires specific measurement. From a public health perspective, the results suggest that addressing affective concerns—rather than solely providing analytic risk data—is crucial for effective communication. The COVID-19 Vaccination Anxiety Scale provides a useful tool for monitoring population-level anxiety and evaluating targeted interventions in Japan. Future studies should examine whether the same structure is replicated in younger and more age-balanced samples and whether the scale can be adapted for use in other settings.

## Introduction

Since 2020, COVID-19 vaccines have been rapidly developed and distributed free of charge in several countries, playing a central role in public health policies. Their effectiveness in preventing severe illness has been confirmed by scientific evidence. For example, a study in the United States reported that the Omicron-variant-specific vaccine (BNT162b2 XBB) had a 62% and 73.3% efficacy in preventing hospitalization and emergency department visits, respectively [1]. Domestic research in Japan has also shown that COVID-19 vaccines (XBB.1.5) can help prevent hospitalization despite their limited effectiveness in preventing infection [2]. Regular vaccination is recommended, however, as its effectiveness may weaken over time [3].

Despite the proven effectiveness and prevalence of vaccines, there remains deep-seated distrust and anxiety regarding their efficacy and safety [4]. While public health discussions often frame this issue primarily in terms of “vaccine hesitancy,” it is crucial to distinguish this behavioral concept from underlying vaccination anxiety. Vaccine hesitancy refers to a broader behavioral tendency to delay or refuse vaccination despite its availability [5], whereas vaccination anxiety refers to the emotional and cognitive distress associated with vaccination [6]. While related, these constructs are not identical. For example, individuals may experience substantial anxiety about vaccination while still choosing to be vaccinated, whereas in other cases, such anxiety may contribute to delay or refusal [7]. This study focuses specifically on vaccination anxiety.

Psychological factors, such as anxiety and distrust, have been identified as key elements associated with lower vaccine acceptance. Lazarus et al. [8] reported that in an international comparative survey of 13,426 people in 19 countries in June 2020, 71.5% of respondents said they would like to be vaccinated if the vaccine was safe and effective. To provide behavioral context, we reviewed later country-level vaccination uptake in the same 19 countries [9]. Across these countries, subsequent vaccination coverage ranged from 33.7% in South Africa and 36.4% in Nigeria to 92.9% in Singapore and 90.1% in China. Notably, some countries with relatively modest early acceptance later achieved high uptake (e.g., Singapore, 67.9% vs. 90.8%), whereas others showed the opposite pattern (e.g., South Africa, 81.6% vs. 35.1%). These descriptive comparisons suggest that early self-reported vaccine acceptance did not map onto later vaccination uptake perfectly, indicating that underlying psychological attitudes are complex and do not fully determine subsequent vaccination behavior.

Although Japan was not included in the Lazarus et al. survey [8], subsequent Japanese data suggest a similar distinction between self-reported attitudes and actual vaccination behavior. According to a domestic survey by Fukunaga [10], prior to April 2021, approximately 30% of respondents reported a lack of trust in the safety and efficacy of the vaccine; importantly, however, about half of this distrustful group still intended to be vaccinated. This suggests a psychological state in which individuals reluctantly choose to receive the vaccine despite harboring significant anxiety. A longitudinal study in Japan found that vaccination intention among the general public increased from 62.1% before vaccine distribution to 72.4% after distribution began [11]. At the population level, vaccine coverage for the primary two-dose regimen later reached 80.4% by December 2022 [12]. Taken together, these findings suggest that concerns or anxiety about vaccination do not necessarily translate into non-vaccination directly. Rather, the relationship between psychological responses and actual vaccine uptake may be shaped by broader social, institutional, and policy contexts. This further supports the importance of examining vaccination anxiety directly, rather than inferring it solely from vaccination behavior or expressed willingness.

Anxiety about vaccination is not unique to COVID-19; it has also been observed in the past with the introduction of influenza and HPV vaccines. According to risk perception theory [13], vaccine-related anxiety can arise when affective, intuitive reactions outweigh analytic, evidence-based assessments of vaccine benefits and adverse-event probabilities at the time of decision making. In such cases, perceived risk may be amplified even when objective risk estimates are low. The intensity and content of such anxiety can vary significantly depending on the social context [14]. The factors specific to COVID-19 vaccine anxiety include, first, the rapid spread and confusion of information in an unprecedented pandemic; second, unfounded anxiety about the new mRNA technology; and third, the inclusion of altruistic motives such as “for the sake of society” and “for the sake of others” in vaccination intentions. These factors are believed to have caused complex and multifaceted psychological reactions. Romate et al. [15] conducted a systematic review and identified fear of infection and severe illness, concerns about vaccine safety and side effects, and anxiety about interactions with preexisting conditions as the main factors associated with COVID-19 vaccine hesitancy. Additionally, trust in vaccines in general, trust in the government and healthcare institutions, beliefs in conspiracy theories, and emotional factors such as health anxiety also significantly influence vaccination anxiety. Cross-national European evidence indicates that conspiracy beliefs are associated not only with lower COVID-19 vaccine uptake but also with weaker support for pandemic-related public health policies [16]. Related work further suggests that COVID-19 conspiracy beliefs are linked to emotionally charged responses such as anger and anxiety [17]. Given the complex interplay among these factors, it is necessary to develop a scale that considers these multifaceted components to accurately assess COVID-19 vaccination anxiety.

Various scales have been developed to capture this multifaceted structure. For example, the Oxford COVID-19 Vaccine Hesitancy Scale developed by Freeman et al. [18] succinctly captures vaccination attitudes and refusal tendencies but does not sufficiently address the content of anxiety. Additionally, its validity has only been verified in relation to the fear of infection, leaving room for further validation. The COVID-19 Vaccine Concerns Scale by Gregory et al. [19] captures concerns specific to COVID-19 but does not address anxiety or fear itself. Therefore, it has a limited ability to capture the diversity of psychological backgrounds. Furthermore, the Multidimensional Vaccine Hesitancy Scale by Balgiu et al. [20] takes a comprehensive approach with eight factors; however, some of these factors are not specific to COVID-19 and instead focus on elements common to general vaccination (cost, inconvenience of vaccination, pain, etc.), limiting its application to COVID-19. Furthermore, there is no standardized scale in Japan, highlighting the need to develop measurement tools tailored to the characteristics of the Japanese population. International surveys have reported comparatively low vaccine confidence in Japan [21], and past vaccine controversies (e.g., the prolonged suspension of proactive HPV vaccine recommendations) [22] may have contributed to persistent safety concerns and distrust. Reviews of COVID-19 vaccine hesitancy in Japan also highlight context-specific themes such as mistrust of institutions and “zero-risk” expectations [23]. Therefore, a validated Japanese-language measure is needed to monitor vaccination-related anxiety and evaluate risk communication and interventions.

To address this gap, this study aimed to develop the COVID-19 Vaccination Anxiety Scale (C19-VAS). We drew upon the scale developed by Al Baseer and Shaheen [6] because it comprehensively and directly captures the emotional distress and multifaceted confidence issues specific to COVID-19 vaccines, overcoming the limitations of previous behavioral or generalized scales. Because the original instrument was designed for university faculty, we revised the item wording to be appropriate and understandable for the general adult population in Japan. We also modified the response format to a five-point Likert scale to improve measurement sensitivity. To examine the construct validity of the C19-VAS, we verified its relationship with the following four existing scales:

The first measure is the Japanese version of the Fear of COVID-19 Scale (FCV-19S-J) [24], which assesses the fear of COVID-19 infection. Individuals with higher levels of fear of the COVID-19 infection are more sensitive to vaccine side effects and efficacy, paradoxically increasing their anxiety about vaccination. Thus, FCV-19S-J and C19-VAS were expected to show a positive correlation (H1).

The second is the Japanese version of the 7C of Vaccination Readiness (7C) [25], which assesses the predictive factors for vaccination intent. As those with higher anxiety about COVID-19 vaccines are likely to have lower readiness for vaccination, it was predicted that the 7C and C19-VAS scores would show a negative correlation (H2).

The third is the Japanese version of the Generic Conspiracist Beliefs Scale (GCBS-J) [26], which assesses general tendencies toward conspiracist beliefs. Individuals with strong conspiratorial thinking tendencies are more likely to harbor distrust regarding the safety of the COVID-19 vaccine and the government’s intentions, leading to heightened vaccination anxiety. Therefore, it was predicted that the GCBS-J and C19-VAS scores would show a positive correlation (H3).

The fourth is the Japanese version of the Perceived Vulnerability to Disease Scale (PVD-J) [27], which assesses awareness of vulnerability to infectious diseases. Individuals with a high perception of vulnerability to infectious diseases are likely to be sensitive to the health effects of vaccines; therefore, it was predicted that the PVD-J and C19-VAS would show a positive correlation (H4).

## Materials and methods

### Participants and procedure

The survey was conducted between April and May 2025, targeting 500 participants aged 18 and over through the web survey service, Freeasy. The de-identified dataset underlying the findings is provided as supporting information (S1 Data). Of the initial 500 participants, we excluded 3 with incomplete responses, 5 physicians or medical professionals, and 72 who failed the Instructional Manipulation Check (IMC) task [28], leaving 420 participants (208 men and 212 women; mean age = 60.33 years, *SD* = 10.70) for the primary analysis. Medical professionals were excluded because their occupational exposure to vaccines, vaccine-related information, and vaccination practices differs substantially from that of the general population. Their inclusion might therefore have introduced a qualitatively different response pattern, making it less appropriate to validate the present scale as a general-population measure.

Additionally, to assess test-retest reliability, these participants were surveyed again after a four-week interval. A total of 356 participants who completed both surveys and successfully passed the follow-up IMC task [28] were included in the test-retest analysis. Table 1 presents the participants’ demographic characteristics.

**Table 1:** Demographic Characteristics of Participants.

Variable	N	(%)	Mean	(SD)
Age	420		60.3	(10.7)
Gender				
Male	208			
Female	212			
Previous COVID-19 Infection				
Yes	119	(28.3)		
No	301	(71.7)		
Chronic diseases				
Yes	104	(24.8)		
No	316	(75.2)		
Living with Family				
Yes	339	(80.7)		
No	81	(19.3)		
Number of Family Members Living Together				
1	98	(23.3)		
2	119	(28.3)		
3	82	(19.5)		
4	32	(7.6)		
5	4	(1.0)		
6	3	(0.7)		
7	1	(0.2)		
Presence or Absence of Children				
Presence	190	(45.2)		
Absence	230	(54.8)		

### Ethical consideration

After the survey’s purpose was presented, the form stated that participation was voluntary and anonymous. Personal information would not be disclosed to third parties. Written informed consent was obtained digitally from all participants; only those who agreed to participate by checking an “I agree” box were able to proceed to the questionnaire. The Tohoku University Graduate School of Education’s Ethics Committee approved this study (ID: 24-1-092).

### Measures

The same questions were asked in the first and second surveys. Table 2 summarizes the details of the FCV-19S-J, 7C, GCBS-J, and PVD-J (construct measured, reference, number of items, and main dimensions), along with our a priori directional hypotheses regarding their associations with the C19-VAS.

**Table 2:** Summary of Measures Used for Construct Validity and A Priori Hypotheses.

Scale (acronym)	Construct measured	Authors/ Year	Number of items	Main dimensions	Expected relation with C19-VAS
Fear of COVID-19 Scale (FCV-19S-J)	Fear of COVID-19 infection	Wakashima et al. (2020) [24]	7	Unidimensional	Positive (H1)
7C of Vaccination Readiness (7C)	Predictive factors for vaccination intent/readiness	Machida et al. (2023) [25]	21	Confidence/ Complacency/ Constraints/ Calculation/ Collective Responsibility/ Compliance/ Conspiracy	Negative (H2)
Generic Conspiracist Beliefs Scale (GCBS-J)	General conspiracist beliefs	Majima and Nakamura (2019) [26]	15	General Conspiracist Beliefs/ Extraterrestrial Conspiracist Beliefs	Positive (H3)
Perceived Vulnerability to Disease (PVD-J)	Perceived vulnerability to infectious disease	Fukukawa et al. (2014) [27]	15	Perceived Infectability/ Germ Aversion	Positive (H4)

Note. H1–H4 denote hypotheses 1–4.

### Sociodemographic variables

Respondents were asked to provide information about their age, gender, pregnancy status, occupation, history of COVID-19 infection, presence of chronic diseases, sources of information on COVID-19, presence of family members living in the same household, and number of family members living in the same household.

### C19-VAS

The scale developed by Al Baseer and Shaheen [6] was translated into Japanese and slightly modified with permission from the original authors. The original items were independently translated into Japanese by the authors, and content validity and naturalness of expression were rigorously reviewed and refined by an expert panel comprising one faculty member and two graduate students specializing in clinical psychology. Participants were instructed as follows (translated from Japanese): “The following questions ask about your views on COVID-19 vaccination. For each item, please indicate the extent to which you feel it applies to you and select the option that best reflects your response.” While Al Baseer and Shaheen [6] used a 3-point scale, a 4-point to 6-point scale was considered the optimal range [29]. Using a 3-point scale or lower may reduce reliability and validity [30]. Therefore, in this study, participants were asked to respond to each item using a 5-point scale ranging from “1 = does not apply” to “5 = applies.” Higher scores indicated greater anxiety about the COVID-19 vaccination. Additionally, when translating and revising the items, we confirmed the content validity with one faculty member with qualifications in clinical psychology and two graduate students specializing in clinical psychology.

### FCV-19S-J

The FCV-19S-J [24] scale consists of 7 items, with responses ranging from “1 = Not at all” to “7 = Strongly agree” on a 7-point scale. In this study, the total score was used for analysis.

### 7C

The 7C [25] scale consists of 21 items, with 3 items for each of the 7 factors, and responses were acquired through a 7-point scale ranging from “1 = Strongly disagree” to “7 = Strongly agree.” In this study, the total score was used for analysis.

### GCBS-J

The GCBS-J [26] scale consists of 15 items across 2 factors, with responses ranging from “1 = Definitely incorrect” to “5 = Definitely correct” on a 5-point scale. In this study, the total score was used for analysis.

### PVD-J

The PVD-J [27] scale consists of 15 items across 2 factors, with responses ranging from “1 = Not at all applicable” to “7 = Very applicable” on a 7-point scale. In this study, the total score was used for analysis.

### Data analysis

Confirmatory factor analysis (CFA) and multiple-group CFA were performed using IBM SPSS AMOS 29. All other analyses were conducted using the IBM SPSS Statistics version 29.

When translating into Japanese, we shifted from a 3-point scale to a 5-point scale and revised some of the items. Consequently, we decided to conduct an exploratory factor analysis (EFA). The data were randomly sampled into two groups: training and testing. First, an EFA was performed on the training data (N = 210) using maximum likelihood estimation and Promax rotation, excluding unnecessary items. CFA was performed on the test data (N = 210). As an additional analysis, multiple-group CFA was conducted to examine gender invariance. To test goodness of fit, we conducted the following analyses: comparative fit index (CFI), root mean square error of approximation (RMSEA), standardized root mean square residual (SRMR), and Akaike information criterion (AIC). The cutoff values for acceptable model fit used in this study were as follows: RMSEA < .10 for acceptable fit and <.06 for good fit; CFI > .90 for acceptable fit and >.95 for good fit; and SRMR < .10 for acceptable fit and <.08 for good fit [31]. Error correlation was assumed to be related to the modified index (MI) if the goodness of fit was inadequate. Reliability was calculated for Cronbach’s alpha coefficients (α) and McDonald’s omega coefficients (ω). Correlations between the C19-VAS score and other measures were established by calculating Pearson’s correlation coefficients. The test-retest correlation coefficient was calculated using ICC (intraclass correlation coefficient, two-way random effects, and absolute agreement).

## Results

### Factor validity

As no items showing ceiling or floor effects were identified, factor analysis was performed on the 30 items of the original C19-VAS. The initial eigenvalues include 14.08, 2.85, 1.75, 1.17, 0.99, in order from the first factor. Based on the scree plot criterion and interpretability of the factors, a three-factor structure was deemed appropriate.

Next, factor analysis was performed, assuming 3 factors; 4 items with factor loadings below 0.40 and 1 item showing double loading were sequentially deleted. The final pattern matrix and inter-factor correlations are shown in Table 3. The cumulative factor contribution rate was 67.59% for the 3 factors across the 25 items. The first factor, “Anxiety Related to Vaccine Confidence,” consists of items such as “I lose confidence in the vaccine’s effectiveness owing to its variety and the different samples tested” and “I am worried about the side effects of the vaccine.” The second factor, “Emotional Symptoms of Anxiety,” comprises items such as “I am overwhelmed by feelings of fear due to excessive thinking about illness and death after vaccination” and “I tremble at the mere thought of vaccinating my family.” The third factor, “Anxiety about Infection after Vaccination,” includes items such as “Even after vaccination, I still dread the thought of being in contact with large numbers of people at work or school” and “I fear attending social gatherings despite being vaccinated.”

**Table 3:** Exploratory Factor Analysis of the C19-VAS.

Item		F1	F2	F3
**F1. Anxiety Related to Vaccine Confidence (α = .96, ω = .96)**				
10	COVID-19ワクチンの種類が多く，検査したサンプルも異なるため，COVID-19ワクチンの有効性に自信が持てない。	**.93**	−.02	−.05
	I lose confidence in the COVID-19 vaccine’s effectiveness due to its variety and the different samples tested.			
9	COVID-19ワクチンを接種することに安心感はない。	**.90**	.002	−.10
	I do not feel a sense of relief from being vaccinated against COVID-19.			
19	COVID-19ワクチンに関する明確なデータがないため，COVID-19ワクチンの有効性を疑っている。	**.89**	.04	−.07
	I doubt the COVID-19 vaccine’s effectiveness due to the lack of clear data about it.			
14	COVID-19ワクチンの副作用が心配だ。	**.84**	−.17	.13
	I am worried about the side effects of the COVID-19 vaccine.			
7	COVID-19ワクチン接種の潜在的な悪影響を心配している。	**.81**	.04	.09
	I am concerned about the potential negative consequences of COVID-19 vaccination.			
21	COVID-19ワクチンの影響に関する情報が錯綜しているため，COVID-19ワクチンの効果に対する信頼を失っている。	**.80**	.16	−.13
	I lose confidence in the COVID-19 vaccine’s effects due to conflicting information about its impact.			
15	ワクチン接種によって起こりうる結果に，医療システムが対処できるかどうか信頼を失っている。	**.79**	.06	.07
	I lose confidence in the healthcare system’s ability to handle the possible consequences of COVID-19 vaccination.			
13	コロナウイルスの新しい変異体を見ると，どんなCOVID-19ワクチンも有用であると信じられなくなる。	**.75**	−.06	−.05
	The new virus variants make me lose faith in the usefulness of any COVID-19 vaccine.			
4	政府のコロナウイルス対策能力を疑っている。	**.73**	.10	−.04
	I doubt the government’s ability to combat the coronavirus.			
26	COVID-19ワクチンの接種による健康被害を心配している。	**.70**	.19	.06
	I fear for my health because of the consequences of the COVID-19 vaccine.			
6	COVID-19ワクチン接種の結果に責任を持つという書類にサインすること自体が恐ろしい。	**.70**	.04	.17
	Signing a form where I take responsibility for any consequences of COVID-19 vaccination is terrifying in itself.			
29	COVID-19ワクチンを接種した後，将来的に健康上の問題が発生することが予想される。	**.69**	.24	−.05
	I anticipate that health problems will occur in the future after COVID-19 vaccination.			
1	COVID-19ワクチンを接種しても，ウイルスへの感染は防げない。	**.69**	−.31	.05
	Vaccination with the COVID-19 vaccine does not prevent infection with the virus.			
25	COVID-19ワクチンが体に何らかの変化をもたらすのではないかと疑っている。	**.68**	.22	.03
	I doubt the COVID-19 vaccine with any change in my body.			
2	COVID-19ワクチン接種により，仕事あるいは学校に支障が出ないか，将来的に心配である。	**.63**	.05	.16
	I am worried that COVID-19 vaccination may cause problems at work or school in the future.			
**F2. Emotional Symptoms of Anxiety (α = .91, ω = .91)**				
28	COVID-19ワクチン接種のことを考えるだけで不安になる。	−.06	**.90**	.03
	I get anxious just thinking about COVID-19 vaccination.			
24	COVID-19ワクチン接種後の病気や死について考えすぎて，恐怖感に打ちひしがれている。	−.39	**.89**	.16
	I am overwhelmed by feelings of fear due to excessive thinking about illness and death after COVID-19 vaccination.			
8	家族の誰かがCOVID-19ワクチンを接種することを考えただけで震える。	.10	**.66**	−.11
	Just thinking about a family member receiving the COVID-19 vaccine makes me tremble.			
27	COVID-19ワクチンを接種することがストレスになっている。	.30	**.63**	−.03
	The idea of COVID-19 vaccination causes me stress.			
16	COVID-19ワクチンの接種義務化についての議論が始まったときからイライラしていた。	.15	**.62**	−.15
	I feel irritable since the beginning of the discussion about mandatory vaccination against COVID-19.			
30	COVID-19ワクチンの有効性に関する数々の噂に心を痛めている。	.13	**.54**	.001
	I am disturbed by the numerous rumors regarding the COVID-19 vaccine’s effectiveness.			
22	COVID-19ワクチンを接種した後に誰かが死んだという話を聞くと，私はストレスを感じる。	.23	**.53**	.11
	When I hear that someone died after receiving the COVID-19 vaccine, I feel stressed.			
**F3. Anxiety about Infection after Vaccination (α = .76, ω = .78)**				
5	COVID-19ワクチンを接種した後でも，職場あるいは学校で大勢の人と接するのは恐ろしい。	.07	−.11	**.89**
	Even after being vaccinated against COVID-19, I am afraid of being in contact with many people at work or school.			
23	COVID-19ワクチンを接種しているにもかかわらず，社交の場に出席するのが怖い。	−.15	.24	**.73**
	Even though I am vaccinated, I am afraid of attending social gatherings.			
3	COVID-19ワクチンを接種したにもかかわらず，コロナウイルスに感染してしまうのではないかと心配している。	.23	−.08	**.58**
	I fear that I might get infected with the virus despite COVID-19 vaccination.			
	Correlation between C19-VAS			
	F1	―	.68	.26
	F2		―	.37
	F3			―

Notes. training data (N = 210); α, Cronbach’s alpha; ω, McDonald’s omega; the numbers in the leftmost column indicate the item order in the C19-VAS as administered in this study. The Japanese text reflects the exact wording used in the survey. The English text shown beneath each item is a corresponding translation provided for reference.

**Fig 1:**
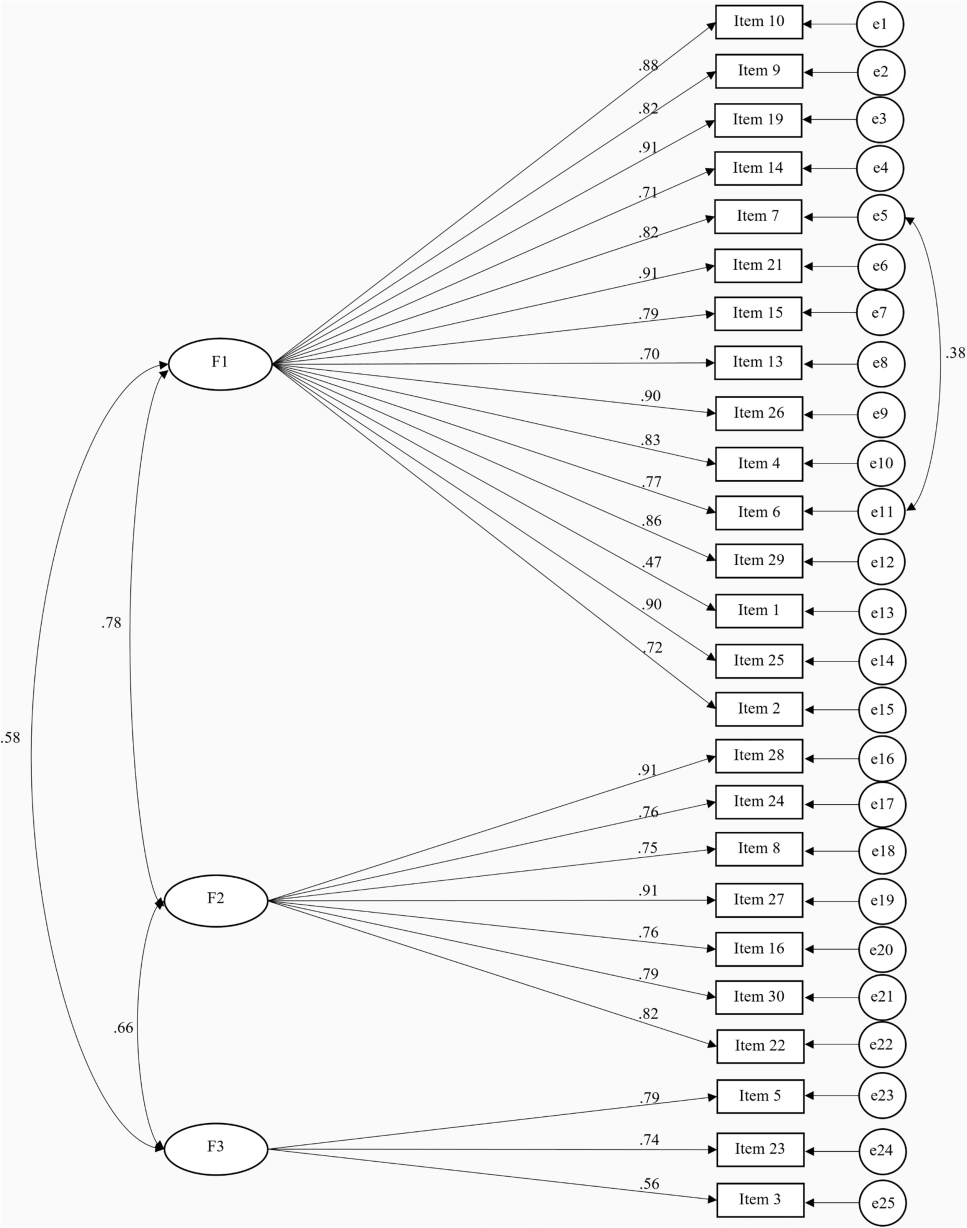
Final three-factor CFA model of the C19-VAS.

Finally, a CFA was performed using the three factors obtained through EFA as latent variables. The results of the CFA of the 3-factor model, with paths drawn from each latent variable to the corresponding items, showed that the model fit indices were χ2 = 781.311, p < .001, df = 272, CFI = .894, RMSEA = .095 [90% CI = .087–.102], SRMR = .073, AIC = 887.311, indicating insufficient model fit. MI was used to improve the model fit. The MI between items 6 and 7 (MI = 28.94) was the highest. Therefore, a within-factor error covariance between items 6 and 7 was included, and the model was modified. The results indicated that the modified model was more acceptable (χ2 = 750.866, p < .001, df = 271, CFI = .901, RMSEA = .092 [90% CI = .084–.100], SRMR = .072, AIC = 858.866) and was used as the final model (Fig 1). Descriptive statistics of the C19-VAS and other scales are presented in Table 4.

**Table 4:** Descriptive Statistics of the Sample’s Responses to the Study Scales.

Variable	Mean	SD	Minimum	Maximum
**C19-VAS**	74.85	19.74	25	125
Anxiety Related to Vaccine Confidence (α = .96, ω = .96)	48.32	13.32	15	75
Emotional Symptoms of Anxiety (α = .91, ω = .91)	17.59	6.18	7	35
Anxiety about Infection after Vaccination (α = .76, ω = .78)	8.94	2.76	3	15
**FCV-19S-J (α = .90, ω = .90)**	15.45	5.09	7	29
**7C**	84.12	14.33	33	131
Confidence (α = .79, ω = .80)	12.00	3.27	3	21
Complacency (α = .86, ω = .86)	13.54	3.53	3	21
Constraints (α = .73, ω = .74)	12.30	3.09	3	21
Calculation (α = .44, ω = .47)	10.24	2.42	3	21
Collective Responsibility (α = .88, ω = .90)	13.12	3.45	3	21
Compliance (α = .75, ω = .76)	10.44	3.06	3	20
Conspiracy (α = .76, ω = .76)	12.47	2.86	3	21
**GCBC-J**	37.17	12.85	15	75
General Conspiracist Beliefs (α = .96, ω = .96)	30.19	10.30	12	60
Extraterrestrial Conspiracist Beliefs (α = .92, ω = .92)	6.97	2.94	3	15
**PVD-J**	62.67	10.59	27	93
Perceived Infectability (α = .84, ω = .83)	26.54	5.99	7	48
Germ Aversion (α = .78, ω = .78)	36.13	7.11	11	56

Notes. α, Cronbach’s alpha; ω, McDonald’s omega.

### Multiple-group CFA

To examine whether the same factor patterns apply to the male and female subsamples, we examined the measurement invariance by gender. Specifically, we confirmed the fit for each gender, then compared four models across datasets: a configuration-invariant model with no equivalence constraints on each dataset; a weak measurement invariance model with equivalence constraints only on factor loadings; a strong measurement invariance model with equivalence constraints on both factor loadings and intercepts; and a strict measurement invariance model with equivalence constraints on both factor loadings, intercepts, and error variables.

Regarding the goodness of fit for each gender, male individuals had χ2 = 559.142, p < .001, df = 271, CFI = .891, RMSEA = .104 [90% CI = .091–.116], SRMR = .080, AIC = 667.142, and female individuals had χ2 = 589.683, p < .001, df = 271, CFI = .859, RMSEA = .104 [90% CI = .092–.115], SRMR = 0.085, AIC = 697.683. Although the CFI criterion was not met, the RMSEA was slightly above the criterion value and the SRMR was below the criterion value for both men and women, indicating a marginally acceptable level of fit. We conducted a multi-population simultaneous analysis by gender for the configuration- and measurement-invariant models. The results showed that the fit of the configuration-invariant model was χ2 = 1148.837, p < .001, df = 542, CFI = .877, RMSEA = .073 [90% CI = .067–.079], SRMR = .080, AIC = 1464.837. The fit of the weak measurement invariance model was χ2 = 1186.602, p < .001, df = 563, CFI = .873, RMSEA = .073 [90% CI = .067–.079], SRMR = .076, AIC = 1460.602. The fit of the strong measurement invariance model was χ2 = 1220.149, p < .001, df = 588, CFI = .871, RMSEA = .072 [90% CI = .066–.078], SRMR = .076, AIC = 1444.149. Finally, the fit of the strict measurement invariance model was χ2 = 1260.177, p < .001, df = 613, CFI = .868, RMSEA = .071 [90% CI = .066–.077], SRMR = .079, AIC = 1434.177. Although the CFI value decreased as the number of equivalence constraints increased, the maximum decrease was only.009, and the RMSEA and SRMR values indicated a good fit. Furthermore, when comparing each model, including the AIC values, the strict measurement invariance model showed the best values among the four models, confirming strict measurement invariance (Table 5). Based on the above, as strict measurement invariance was confirmed for gender, the same three-factor structure was applied to construct the scale regardless of gender.

**Table 5:** Measurement Invariance of Model among Gender Groups for C19-VAS.

	χ2	df	CFI	RMSEA	SRMR	AIC
Configural model	1148.837	542	.877	.073	.080	1464.837
Metric invariance model	1186.602	563	.873	.073	.076	1460.602
Scalar invariance model	1220.149	588	.871	.072	.076	1444.149
Strict invariance model	1260.177	613	.868	.071	.079	1434.177

Notes. test data (N = 210); CFI, comparative fit index; RMSEA, root mean square approximation; SRMR, standardized root mean square residual; and AIC, Akaike information criterion.

### Examination of gender differences

To examine gender differences in COVID-19 vaccine anxiety, a t-test (two-tailed) was conducted on 420 participants at Time 1. The results showed a significant gender difference (t (418) = 2.34, p = .02, d = .23). This indicates that women had higher total scores on the scale than men.

### Reliability

Cronbach’s α and McDonald’s ω were calculated for the subscales of C19-VAS using data from 420 participants at Time 1. The first factor had α = .96 and ω = .96; the second factor had α = .91 and ω = .91; and the third factor had α = .76 and ω = .78, demonstrating sufficient internal consistency. Additionally, the ICC between Time 1 and Time 2 for “Anxiety Related to Vaccine Confidence” was.94 (95% CI [.92–.96]), “Emotional Symptoms of Anxiety” was.91 (95% CI [.89–.93]), and “Anxiety about Infection after Vaccination” was.81 (95% CI [.77–.85]).

### Construct validity

To verify the construct validity of the C19-VAS, we performed a correlation analysis with the total scores of the FCV-19S-J, 7C, GCBS-J, and PVD-J at Time 1. When examining the correlation coefficients between the C19-VAS total score and FCV-19S-J, 7C, GCBS-J, and PVD-J, there was a moderate to strong positive correlation with FCV-19S-J (r = .44, p < .001), a strong negative correlation with 7C (r = −.59, p < .001), a strong positive correlation with GCBS-J (r = .55, p < .001), and a small to moderate positive correlation with PVD-J (r = .19, p < .001). As summarized in Table 6, H1–H4 were supported.

**Table 6:** Construct Validity: Correlations between the C19-VAS and Validation Measures.

Hypothesis	Validation measure	Predicted direction	r	p-value	Supported?
H1	FCV-19S-J	positive	.44	<.001	Yes
H2	7C	negative	−.59	<.001	Yes
H3	GCBS-J	positive	.55	<.001	Yes
H4	PVD-J	positive	.19	<.001	Yes

## Discussion

This study aimed to develop the C19-VAS to measure COVID-19 vaccine anxiety in Japanese adults. Factor analysis revealed that the C19-VAS was constructed with 3 factors and 25 items: “Anxiety Related to Vaccine Confidence,” “Emotional Symptoms of Anxiety,” and “Anxiety about Infection after Vaccination.” These three factors differ from those developed by Al Baseer and Shaheen [6]. This is likely owing to the change from a 3-point scale to a 5-point scale and some revisions to the scale items. The scale developed by Al Baseer and Shaheen [6] was designed for university faculty, whereas the C19-VAS was revised for application in the general adult population. Therefore, the factor structure assumed by Al Baseer and Shaheen [6] was not observed.

In the CFA, allowing an error correlation between items 6 and 7 improved model fit to an acceptable, although not ideal, level. Conceptually, both items refer to broad anticipated harm arising from COVID-19 vaccination itself: item 7 concerns the potential negative consequences of vaccination in general, whereas item 6 refers to fear elicited by assuming personal responsibility for such consequences. In this sense, these two items appear to share a residual component related to generalized concern about adverse consequences, beyond the broader latent factor of “Anxiety Related to Vaccine Confidence.” By contrast, most of the other items in this factor refer to more specific sources of reduced confidence, such as side effects, insufficient data, conflicting information, or distrust in the government and in the healthcare system. We, therefore, considered the correlated residual theoretically and conceptually plausible, rather than a purely data-driven adjustment.

Nonetheless, the fit indices should be interpreted with caution. In the single-group CFA, although the final three-factor model improved in the initial solution, the CFI values were not uniformly strong, suggesting some residual item redundancy. For example, items 10 and 19 were semantically similar and showed a particularly high inter-item correlation (r = .83), suggesting that some overlap in item content may still remain. However, rather than relying on a single fit index alone, we retained the three-factor solution based on multiple considerations: the scree plot, interpretability and conceptual distinctiveness of the three domains, the fact that the final 25-item solution still explained a substantial proportion of the total variance, and adequate internal consistency of the resulting factors.

A multiple-group CFA was also conducted to test measurement invariance by gender. Across models, the absolute CFI values were slightly below the conventional.90 criterion, whereas RMSEA and SRMR were within or close to commonly used benchmark values. Therefore, these results should not be interpreted as indicating optimal fit, but rather, as a marginally acceptable fit. Nevertheless, the decrements in fit indices across the configural, metric, scalar, and strict invariance models were small, and strict invariance showed the most favorable AIC among the tested models. According to Chen [32], a ∆CFI of ≤.010 indicates that the invariance assumption is not substantially violated. In our analysis, the total decrement from the configural to the strict invariance model was only.009. Therefore, rather than judging invariance based on the absolute CFI value alone, it was judged based on the overall pattern of evidence, including the relative stability of RMSEA and SRMR and the small change in CFI across increasingly constrained models. Taken together, these findings suggest that the three-factor model is reasonably comparable across both men and women, while also indicating that some refinement of item wording or scale length may further improve model fit in future studies.

Next, the results of the gender difference analysis showed that women had higher COVID-19 vaccination anxiety than men. The results were consistent with those of Sekizawa et al. [33], who showed that women tended to be negative about COVID-19 vaccination. Women experience more side effects from the COVID-19 vaccine owing to hormonal differences between men and women [34], which may have contributed to gender differences in anxiety about COVID-19 vaccination. Additionally, women generally have higher sensitivity to COVID-19-related risks [35], and such personality traits may also influence gender differences in anxiety.

Internal consistency and test-retest reliability were also examined. The α and ω coefficients in C19-VAS returned sufficient values. Test-retest reliability also showed sufficient values for all three factors, confirming test-retest reliability. Subsequently, to verify construct validity, a correlation analysis was conducted between the C19-VAS at Time 1 and the total scores on the FCV-19S-J, 7C, GCBS-J, and PVD-J. The results showed positive correlations with FCV-19S-J, GCBS-J, and PVD-J, and a negative correlation with 7C.

The association with GCBS-J is particularly noteworthy. Similar associations have been reported both beyond the COVID-19 context and across diverse national settings. Health psychology studies have shown that conspiracist beliefs are associated with general anti-vaccination attitudes [36,37] and lower HPV vaccination intention [38]. These findings suggest that a broader conspiracist worldview may predispose individuals to interpret vaccines as threatening, untrustworthy, or harmful. Political science studies have shown comparable associations in cross-national COVID-19 research. For example, lower COVID-19 vaccine acceptance has been associated with lower trust in authorities and scientists and stronger conspiratorial thinking across multiple countries [39,40]. Related comparative research has also shown that conspiracy and misinformation beliefs are linked to emotional states and trust in information sources across countries [41]. In addition, Miller’s work supports the view that specific COVID-19 conspiracy beliefs are embedded in a broader conspiracist worldview shaped by distrust and uncertainty [42,43]. More broadly, Filsinger and Freitag showed that vaccination attitudes can become a source of affective polarization across multiple European countries [44]. Taken together, these findings suggest that the present association between general conspiracist beliefs and COVID-19 vaccination anxiety in Japanese adults is consistent with broader evidence indicating that conspiracist thinking may shape not only vaccine acceptance but also the affective meaning attached to vaccination.

The correlation coefficient with PVD-J was lower than those with other scales, which was likely due to differences in the factors of PVD-J, namely, “perceived infectability” and “germ aversion.” When the correlation between each factor and C19-VAS score was examined, a significant correlation (r = .20, p < .001) was confirmed with the latter but no correlation (r = .09, p = .06) was confirmed with the former. This suggests that perceived infectability is not associated with COVID-19 vaccine anxiety, whereas perceived discomfort in situations where pathogens are likely to be encountered is associated with COVID-19 vaccine anxiety. Duncan et al. [45] distinguished between “perceived infectability,” which is a response based on rational judgment, and “germ aversion,” which is a response based on intuitive judgment. Items such as “I don’t want to wear secondhand clothes because I don’t know who last wore them” reflect “germ aversion” and measure anxious tendencies toward infection risks that lack empirical basis [27]. Regarding the COVID-19 vaccine, providing unfounded conspiracy theories or misinformation increases hesitancy toward vaccination [46]. Many conspiracy theories and rumors exploit intuitive reactions rather than theoretical evidence of adverse events. Therefore, COVID-19 vaccine anxiety and “germ aversion” share a similar cognitive tendency to avoid risks through immediate judgments based on weak evidence, which may explain the observed correlation. Meanwhile, “perceived infectability” is a response based on rational judgment [45]. Therefore, no significant correlation was demonstrated between “perceived infectability” and C19-VAS. From a public health communication perspective, this finding implies that simply providing analytic information about infection risk or vaccine effectiveness may be insufficient for reducing vaccination anxiety. Communication strategies also need to address affective concerns, such as fear, disgust, and anticipated discomfort, in a transparent and reassuring manner. Further investigation is necessary in this regard. Based on these results, the C19-VAS was considered to have sufficient reliability and validity.

This study has some limitations. First, healthcare workers were excluded from the analysis. Because healthcare workers received COVID-19 vaccines earlier and more frequently than the general population, their risk perceptions and psychological responses may differ. Consequently, the factor structure of the C19-VAS, which was developed for the general adult population, might not fully apply to them. Future research should investigate the scale’s validity in healthcare settings or develop a scale tailored to this population. Second, this study did not include questions about vaccination history before taking the COVID-19 vaccine. The presence or absence of vaccination experience may influence attitudes toward the vaccine and anxiety. In the future, it will be necessary to collect detailed information on vaccination history, such as the number of doses administered and the presence or absence of adverse reactions, and examine their association with vaccination anxiety. Third, the analyzed sample was skewed toward older adults, which limits the generalizability of the findings to younger Japanese adults. Previous studies in Japan have suggested that younger adults are more likely to show COVID-19 vaccine hesitancy, whereas older age is associated with greater willingness to vaccinate and more frequent booster uptake [47]. Therefore, this age imbalance may have influenced not only the overall level of reported vaccination anxiety, but also the relative salience of specific psychological factors reflected in the factor structure. Accordingly, the findings should be interpreted primarily as evidence from a relatively older adult sample, and future studies should examine whether the same factor structure and psychometric properties are replicated in younger and more age-balanced populations. Fourth, because the survey was conducted in 2025, the salience of COVID-19 vaccination may have varied across respondents depending on recent experiences, such as infection history or exposure to COVID-19-related information. Although this study assessed current thoughts and feelings about COVID-19 vaccination, rather than a retrospective recall of the early pandemic period, we did not directly model topic salience in the analyses. To address this limitation, future studies should examine how recent exposure, infection history, and related contextual factors may influence C19-VAS responses.

## Conclusions

This study clarified the factor structure of the C19-VAS, which demonstrated good internal consistency and test–retest reliability. Although the acute phase of the pandemic has passed, COVID-19 vaccination remains an ongoing public health issue in Japan in 2025, with routine annual vaccination for older adults and high-risk groups, and voluntary vaccination options outside the routine program [48]. Globally, the WHO continues to recommend that countries offer COVID-19 vaccination and notes sustained SARS-CoV-2 evolution, which may necessitate updates to vaccine composition [49].

Importantly, vaccination anxiety is not fully captured by vaccination uptake alone: Affective reactions can coexist with vaccine acceptance and may fluctuate with policy, information environments, and trust. This is consistent with risk-perception frameworks distinguishing “risk as feelings” from analytic risk appraisal [13]. The C19-VAS provides a brief, validated tool to quantify this psychological component, enabling population monitoring, the identification of groups with heightened anxiety, and the evaluation of risk communication strategies and interventions in Japan. While the scale was validated for Japan and the COVID-19 context, it also offers a framework for future work in other settings with appropriate cultural adaptation and psychometric revalidation.

## Supporting information

S1 File C19-VAS. (DOCX)

S2 File C19-VAS_English Translation. (DOCX)

S1 Data Anonymized data set. (XLSX)
