# Supplementary material for: Development of a COVID-19 Vaccination Anxiety Scale to measure COVID-19 vaccine anxiety in Japanese adults
Source: PLoS One. 2026 Jul 7;21(7):e0330146. doi: 10.1371/journal.pone.0330146 (PMC13340786; doi:10.1371/journal.pone.0330146)
Supplement: S1 File — (DOCX) [file pone.0330146.s001.docx]

COVID-19 Vaccination Anxiety Scale

以下の項目について，“あてはまらない”，“あまりあてはまらない”，“どちらともいえない”，“ややあてはまる”，“あてはまる”のうち，あてはまる数字に○をつけてください。

|  |  | あてはまらない | あまりあてはまらない | どちらともいえない | ややあてはまる | あてはまる |
| --- | --- | --- | --- | --- | --- | --- |
| 1 | COVID-19ワクチンを接種しても，ウイルスへの感染は防げない。 | 1 | 2 | 3 | 4 | 5 |
| 2 | COVID-19ワクチン接種により，仕事あるいは学校に支障が出ないか，将来的に心配である。 | 1 | 2 | 3 | 4 | 5 |
| 3 | COVID-19ワクチンを接種したにもかかわらず，コロナウイルスに感染してしまうのではないかと心配している。 | 1 | 2 | 3 | 4 | 5 |
| 4 | 政府のコロナウイルス対策能力を疑っている。 | 1 | 2 | 3 | 4 | 5 |
| 5 | COVID-19ワクチンを接種した後でも，職場あるいは学校で大勢の人と接するのは恐ろしい。 | 1 | 2 | 3 | 4 | 5 |
| 6 | COVID-19ワクチン接種の結果に責任を持つという書類にサインすること自体が恐ろしい。 | 1 | 2 | 3 | 4 | 5 |
| 7 | COVID-19ワクチン接種の潜在的な悪影響を心配している。 | 1 | 2 | 3 | 4 | 5 |
| 8 | 家族の誰かがCOVID-19ワクチンを接種することを考えただけで震える。 | 1 | 2 | 3 | 4 | 5 |
| 9 | COVID-19ワクチンを接種することに安心感はない。 | 1 | 2 | 3 | 4 | 5 |
| 10 | COVID-19ワクチンの種類が多く，検査したサンプルも異なるため，COVID-19ワクチンの有効性に自信が持てない。 | 1 | 2 | 3 | 4 | 5 |
| 11 | コロナウイルスの新しい変異体を見ると，どんなCOVID-19ワクチンも有用であると信じられなくなる。 | 1 | 2 | 3 | 4 | 5 |
| 12 | COVID-19ワクチンの副作用が心配だ。 | 1 | 2 | 3 | 4 | 5 |
| 13 | ワクチン接種によって起こりうる結果に，医療システムが対処できるかどうか信頼を失っている。 | 1 | 2 | 3 | 4 | 5 |
| 14 | COVID-19ワクチンの接種義務化についての議論が始まったときからイライラしていた。 | 1 | 2 | 3 | 4 | 5 |
| 15 | COVID-19ワクチンに関する明確なデータがないため，COVID-19ワクチンの有効性を疑っている。 | 1 | 2 | 3 | 4 | 5 |
| 16 | COVID-19ワクチンの影響に関する情報が錯綜しているため，COVID-19ワクチンの効果に対する信頼を失っている。 | 1 | 2 | 3 | 4 | 5 |
| 17 | COVID-19ワクチンを接種した後に誰かが死んだという話を聞くと，私はストレスを感じる。 | 1 | 2 | 3 | 4 | 5 |
| 18 | COVID-19ワクチンを接種しているにもかかわらず，社交の場に出席するのが怖い。 | 1 | 2 | 3 | 4 | 5 |
| 19 | COVID-19ワクチン接種後の病気や死について考えすぎて，恐怖感に打ちひしがれている。 | 1 | 2 | 3 | 4 | 5 |
| 20 | COVID-19ワクチンが体に何らかの変化をもたらすのではないかと疑っている。 | 1 | 2 | 3 | 4 | 5 |
| 21 | COVID-19ワクチンの接種による健康被害を心配している。 | 1 | 2 | 3 | 4 | 5 |
| 22 | COVID-19ワクチンを接種することがストレスになっている。 | 1 | 2 | 3 | 4 | 5 |
| 23 | COVID-19ワクチン接種のことを考えるだけで不安になる。 | 1 | 2 | 3 | 4 | 5 |
| 24 | COVID-19ワクチンを接種した後，将来的に健康上の問題が発生することが予想される。 | 1 | 2 | 3 | 4 | 5 |
| 25 | COVID-19ワクチンの有効性に関する数々の噂に心を痛めている。 | 1 | 2 | 3 | 4 | 5 |
