# Supplementary material for: Development of a COVID-19 Vaccination Anxiety Scale to measure COVID-19 vaccine anxiety in Japanese adults
Source: PLoS One. 2026 Jul 7;21(7):e0330146. doi: 10.1371/journal.pone.0330146 (PMC13340786; doi:10.1371/journal.pone.0330146)
Supplement: S2 File — (DOCX) [file pone.0330146.s002.docx]

COVID-19 Vaccination Anxiety Scale (English Translation)

For each of the following items, please circle the number that best describes your agreement: "Strongly Disagree," "Disagree," "Neutral," "Agree," or "Strongly Agree."

|  |  | Strongly Disagree | Disagree | Neutral | Agree | Strongly Agree |
| --- | --- | --- | --- | --- | --- | --- |
| 1 | Vaccination with the COVID-19 vaccine does not prevent infection with the virus. | 1 | 2 | 3 | 4 | 5 |
| 2 | I am worried that COVID-19 vaccination may cause problems at work or school in the future. | 1 | 2 | 3 | 4 | 5 |
| 3 | I fear that I might get infected with the virus despite COVID-19 vaccination. | 1 | 2 | 3 | 4 | 5 |
| 4 | I doubt the government’s ability to combat the coronavirus. | 1 | 2 | 3 | 4 | 5 |
| 5 | Even after being vaccinated against COVID-19, I am afraid of being in contact with many people at work or school. | 1 | 2 | 3 | 4 | 5 |
| 6 | Signing a form where I take responsibility for any consequences of COVID-19 vaccination is terrifying in itself. | 1 | 2 | 3 | 4 | 5 |
| 7 | I am concerned about the potential negative consequences of COVID-19 vaccination. | 1 | 2 | 3 | 4 | 5 |
| 8 | Just thinking about a family member receiving the COVID-19 vaccine makes me tremble. | 1 | 2 | 3 | 4 | 5 |
| 9 | I do not feel a sense of relief from being vaccinated against COVID-19. | 1 | 2 | 3 | 4 | 5 |
| 10 | I lose confidence in the COVID-19 vaccine’s effectiveness due to its variety and the different samples tested. | 1 | 2 | 3 | 4 | 5 |
| 11 | The new virus variants make me lose faith in the usefulness of any COVID-19 vaccine. | 1 | 2 | 3 | 4 | 5 |
| 12 | I am worried about the side effects of the COVID-19 vaccine. | 1 | 2 | 3 | 4 | 5 |
| 13 | I lose confidence in the healthcare system’s ability to handle the possible consequences of COVID-19 vaccination. | 1 | 2 | 3 | 4 | 5 |
| 14 | I feel irritable since the beginning of the discussion about mandatory vaccination against COVID-19. | 1 | 2 | 3 | 4 | 5 |
| 15 | I doubt the COVID-19 vaccine’s effectiveness due to the lack of clear data about it. | 1 | 2 | 3 | 4 | 5 |
| 16 | I lose confidence in the COVID-19 vaccine’s effects due to conflicting information about its impact. | 1 | 2 | 3 | 4 | 5 |
| 17 | When I hear that someone died after receiving the COVID-19 vaccine, I feel stressed. | 1 | 2 | 3 | 4 | 5 |
| 18 | Even though I am vaccinated, I am afraid of attending social gatherings. | 1 | 2 | 3 | 4 | 5 |
| 19 | I am overwhelmed by feelings of fear due to excessive thinking about illness and death after COVID-19 vaccination. | 1 | 2 | 3 | 4 | 5 |
| 20 | I doubt the COVID-19 vaccine with any change in my body. | 1 | 2 | 3 | 4 | 5 |
| 21 | I fear for my health because of the consequences of the COVID-19 vaccine. | 1 | 2 | 3 | 4 | 5 |
| 22 | The idea of COVID-19 vaccination causes me stress. | 1 | 2 | 3 | 4 | 5 |
| 23 | I get anxious just thinking about COVID-19 vaccination. | 1 | 2 | 3 | 4 | 5 |
| 24 | I anticipate that health problems will occur in the future after COVID-19 vaccination. | 1 | 2 | 3 | 4 | 5 |
| 25 | I am disturbed by the numerous rumors regarding the COVID-19 vaccine’s effectiveness. | 1 | 2 | 3 | 4 | 5 |
